# The efficacy of Fire dragon cupping combined with Shaofu Zhuyu decoction in treating primary dysmenorrhea with cold coagulation and blood stasis

**DOI:** 10.12669/pjms.41.10.12648

**Published:** 2025-10

**Authors:** Zijiao Yang, Shaojing Zeng, Xiaohua Lin, Zhifang Qiao, Wenhui Bian, Yujiao Zhang

**Affiliations:** 1 Zijiao Yang Department of Gynecology, Hebei Provincial Hospital of Chinese Medicine, The First Affiliated Hospital of Hebei University of Chinese Medicine, Shijiazhuang, Hebei Province 050011, P.R. China; 2 Shaojing Zeng, Department of Endocrinology,Hebei Provincial People’s Hospital, Shijiazhuang, Hebei, China; 3 Xiaohua Lin Department of Gynecology, Hebei Provincial Hospital of Chinese Medicine, The First Affiliated Hospital of Hebei University of Chinese Medicine, Shijiazhuang, Hebei Province 050011, P.R. China; 4 Zhifang Qiao, Hebei Provincial Hospital of Chinese Medicine, The First Affiliated Hospital of Hebei University of Chinese Medicine, Shijiazhuang, Hebei Province 050011, P.R. China; 5 Wenhui Bian Department of Gynecology, Hebei Provincial Hospital of Chinese Medicine, The First Affiliated Hospital of Hebei University of Chinese Medicine, Shijiazhuang, Hebei Province 050011, P.R. China; 6 Yujiao Zhang, Traditional Chinese Medicine Nursing Clinic, Hebei Provincial Hospital of Chinese Medicine, The First Affiliated Hospital of Hebei University of Chinese Medicine, Shijiazhuang, Hebei Province 050011, P.R. China

**Keywords:** Cold coagulation and blood stasis, Fire Dragon Cupping, Primary dysmenorrhea, Shaofu Zhuyu Decoction

## Abstract

**Objective:**

To evaluate the efficacy of Fire Dragon Cupping (FDC) combined with Shaofu Zhuyu Decoction (SFZYD) in the treatment of primary dysmenorrhea (PD) with cold coagulation and blood stasis (CCBS).

**Methodology:**

A retrospective case-control analysis was conducted at the Department of Gynecology, Hebei Provincial Hospital of Traditional Chinese Medicine (TCM). All participants were selected from a registry of PD CCBS patients who received treatment between October 2022 to May 2024. Patients were retrospectively assigned according to the treatment received to either the SFZYD group or the FDC + SFZYD group. The primary outcome of interest was the TCM syndrome score. The secondary outcomes included changes in visual analogue scale (VAS), uterine artery hemodynamic status, endothelin-1 (ET-1) and nitric oxide (NO) levels.

**Results:**

This study included a total of 112 patients, 54 patients in the SFZYD group and 58 patients in the FDC & SFZYD group. After treatment, the TCM syndrome score and VAS score in the PDC & SFZYD group were significantly lower than those in the SFZYD group (*P*<0.05); The pulsatility index (PI) and the resistance index (RI) in the FDC & SFZYD group were both lower than those in the SFZYD group (*P*<0.05); Compared with SFZYD alone, ET-1 decreased and NO increased in the FDC + SFZYD group (*P*<0.05).

**Conclusions:**

The combination of FDC and SFZYD can more effectively reduce the pain level of PD patients with CCBS, alleviate clinical symptoms, regulate the hemodynamic status of uterine arteries and the levels of ET-1 and NO than SFZYD alone.

## INTRODUCTION

Primary dysmenorrhea (PD) is a common gynecological problem that typically occurs shortly following the onset of puberty but may also persist into adulthood.^1^ PD may be accompanied by varying degrees of abdominal bloating, pain, vomiting, cold hands and feet and other symptoms.^1^,^2^ The condition is more common in women with weak and cold constitutions and insufficiency of yang-qi. Exposure to cold may impair the circulation of qi and blood, contributing to blood stasis. According to Traditional Chinese Medicine (TCM), frequent consumption of raw and cold foods, wearing clothing that reveals the navel and prolonged exposure to cold environments may lead to the invasion of cold air into the body, thereby causing cold coagulation and blood stasis (CCBS).^3^,^4^ Currently, clinical interventions for dysmenorrhea are often implemented through adjusting lifestyle habits, psychological counseling and administering contraceptives and nonsteroidal anti-inflammatory drugs.^5^ While these measures can alleviate clinical symptoms to a certain extent, the overall effect is not satisfactory and the disease recurrence rate is high after discontinuation of medication.^6^,^7^ Therefore, it is necessary to find new treatment strategy suitable for PD patients with CCBS.

Although Western medicine for PD may achieve a good immediate analgesic effect, its overall efficiency is questionable due to the unknown etiology of the disease and drug resistance that often develops after long-term repeated use.^4^,^6^,^7^ In recent years, TCM treatment of PD has received more attention, as it is associated with fewer side effects.^3^,^8^ TCM categorizes dysmenorrhea as “menstrual pain” and other types and divides them into different syndrome types based on their clinical manifestations, with CCBS recognized as one of the most common TCM syndromes.^3^,^4^,^9^

Fire Dragon Cupping (FDC) is a novel Traditional Chinese Medicine (TCM) therapy that uses massage, scraping, moxibustion, kneading, ironing and acupoint stimulation.^10^,^11^ The cup rim is designed to permit sliding (walking-cupping), scraping, and acupoint stimulation. Up to three 3-cm moxa pillars can be placed inside the cup to deliver heat for cold-dispelling and stasis-resolving effects.^11^

Shaofu Zhuyu Decoction (SFZYD), a prescription originating from “Correction of Errors in Medical Classics” compiled by Qing-ren Wang during the Qing dynasty, has been used in clinics to treat CCBS syndrome of gynecological diseases, such as PD, for over 250 years.^12^,^13^

However, there is still limited evidence on the efficacy of combining FDC and SFZYD for treating patients with PD and CCBS. This retrospective analysis aimed to clarify the intervention value of the combination of FDC and SFZYD in treating this group of PD patients.

## METHODOLOGY

This retrospective case-control analysis, conducted in the Department of Gynecology at Hebei Provincial Hospital of TCM, included clinical data from patients with PD and CCBS who received treatment between October 2022 to May 2024. According to the treatment records, patients receiving SFZYD treatment alone were retrospectively included in the SFZYD group, while patients receiving FDC & SFZYD therapy were included in the FDC & SFZYD group. Treatment choice reflected routine clinical practice and was determined primarily by patient preference, physician recommendation, and the patient’s acceptance of each intervention; no randomization or allocation concealment was applied in this retrospective registry.

### Ethical approval:

The Ethics Committee of Hebei Provincial Hospital of TCM has approved the study protocol (No. HBZY2020-KY-024-01, Date: April 23th 2021). All procedures involving human subjects complied with the 1964 Helsinki Declaration and its subsequent amendments or equivalent ethical standards. Due to the retrospective nature of the study, the Ethics Committee of Hebei Provincial Hospital of TCM has waived informed consent. All data were securely stored and kept confidential throughout the entire research process.

PD was defined as dysmenorrhea in the absence of pelvic organic disease on ultrasound and gynecologic examination. According to the Clinical Terminology of TCM Diagnosis and Treatment (National Administration of TCM, 1997), the diagnostic criteria of the CCBS syndrome were as follows:^14^

### Main symptoms:

Lower abdominal pain relieved due to heat before or during menstruation, fear of cold, liking warmth and aversion to stress.

### Concurrent symptoms:

a) Low blood volume, or poor menstruation, or late menstruation. b) Purple menstrual blood with blood clots, the lower abdominal pain is relieved after the blood clots are discharged. c) Pale complexion, aversion to cold and icy limbs. Tongue and pulse: Dark purple tongue or petechial tongue, white coating, slender or deep and tight pulse, or deep and slow and uneven pulse.

The diagnosis of CCBS syndrome was made if the patient had two or more concurrent symptoms, combined with tongue and pulse. All CCBS patients were diagnosed by a chief physician of TCM. In this retrospective registry, CCBS was recorded as a single syndrome category; no standardized quantitative subtype classification (e.g., predominantly cold vs. predominantly blood stasis) was applied at enrollment.

### Inclusion criteria:

- Meet the diagnostic criteria for PD.^1^
- The TCM syndrome classification is CCBS.^14^
- Dysmenorrhea persists for at least three months and is between the ages of 18 and 35.
- Healthy women; 5) VAS score ≥ 4; 6) Complete clinical data.

### Exclusion criteria:

- Dysmenorrhea caused by endometriosis, adenomyosis, etc.
- Patients with renal and liver dysfunction.
- Patients with hematological disorders.
- Patients with acute or chronic infections.
- Patients who have been taking contraceptive pills, hormone drugs, or painkillers within the past three months.
- Patients who have used oral or topical TCM preparations within three months.
- Breastfeeding and pregnant women.

### Intervention methods:

### SFZYD treatment:

SFZYD decoction was provided by the TCM Pharmacy of Hebei Provincial Hospital of TCM. The specific prescription is: Trogopterori Faeces (Wulingzhi) 6g, Cinnamomi cortex (Rougui) 3g, Angelicae sinensis radix (Dangui) 9g, Chuanxiong rhizoma (Chuanxiong) 9g, Corydalis rhizoma (Yanhusuo) 9g, Paeoniae radix rubra (Chishao) 6g, Zingiberis rhizoma (Ganjiang) 6g, Foeniculi fructus (Xiaohuixiang) 3g, Typhae pollen (Puhuang) 9g. Oral administration: one dose per day, divided into morning and evening sessions. The medication is initiated orally four days before menstruation, for seven consecutive days, for three menstrual cycles.

### FDC:

FDC treatment was provided in addition to SFZYD decoction. The patient was placed in a lateral position, exposing the waist and back while keeping the abdomen warm. Essential oil was applied evenly on the back. Moxa pillars were ignited in the cup. Patients reported comfortable warmth without pain. After approximately two minutes, adequate heat penetration was achieved; the cup was then moved along the bilateral bladder meridian with standardized repetitions. Finally, the cupping treatment was enhanced at the baliao acupoint (entwisting, kneading, pointing and vibrating) for three to five minutes. The entire cupping treatment of the back lasted approximately 15 to 20 minutes. During the procedure, the participants’ mental changes were constantly evaluated to ensure timely communication. Any adverse reactions, such as erythema and blisters, were recorded. Adverse events were captured in routine clinical notes; however, standardized grading (e.g., CTCAE) and session-level frequency logs were not uniformly implemented in this retrospective dataset.

Subsequently, the cup was moved along the abdominal midline for 10 standardized passes. Bimanual pinching and kneading were performed five times in both directions, and acupoint pressure/cupping was applied until mild warmth and erythema were observed. Then, the lower abdomen was massaged with vibration for 20 minutes. The abdomen was massaged horizontally 10 times. Treatment was done 3-5 days after the end of menstruation, once every four days, until five days before menstruation, for a total of three menstrual cycles. All included patients completed at least one full treatment cycle according to routine practice; however, session-by-session attendance and standardized completion rates across all three cycles were not consistently documented in the medical records, precluding calculation of uniform adherence metrics.

### Safety:

No serious adverse events were recorded in the medical charts. A small number of patients experienced mild, transient erythema or localized warmth at the cupping sites, all of which resolved spontaneously without intervention. No burns, syncope, or treatment-related hospitalizations were documented. Because adverse events were not captured with standardized grading and session-by-session frequency logs were inconsistently documented, the exact incidence rates could not be calculated for the entire cohort.

### Herbal materials and decoction standardization:

All crude herbs of Shaofu Zhuyu Decoction (SFZYD) were sourced from the GMP-certified hospital TCM pharmacy. Each crude drug was authenticated independently by two senior pharmacognosists according to the Pharmacopoeia of the People’s Republic of China (latest edition in use at the pharmacy). Voucher samples and procurement records were archived (voucher IDs available upon request). The decoction was prepared under a written standard operating procedure (SOP): herbs were weighed according to the formula, inspected, rinsed, and soaked in purified water (approximately 8–10 × w/v) for \~30 minutes; the mixture was decocted twice (about 30 minutes for the first decoction and 20 minutes for the second), filtrates were combined, and the final volume was adjusted to a daily dose divided into morning and evening administrations. The decoction was dispensed fresh daily (or stored at 2–8 °C for ≤24 h if needed) and administered from 4 days before menstruation for seven consecutive days per cycle, for three cycles. Batch-to-batch consistency was ensured at two levels. First, crude herb QC included organoleptic/microscopic identification and TLC assays per pharmacopoeial monographs, moisture/ash limits, and routine checks for restricted contaminants. Second, the pharmacy implemented UPLC/HPLC fingerprinting and quantification of sentinel marker compounds to monitor chemical consistency across production periods. Typical markers included ferulic acid (Angelica sinensis/Chuanxiong), paeoniflorin (Paeoniae radix rubra), cinnamaldehyde (Cinnamomi cortex), 6-gingerol (Zingiberis rhizoma), and alkaloids from Corydalis rhizoma (e.g., tetrahydropalmatine). Batch similarity was evaluated against a reference fingerprint using the pharmacy’s validated workflow. Because this was a retrospective study, comprehensive batch-level chromatograms were not fully archived within our analytic dataset. The QC workflow is therefore described here, and future prospective studies will be pre-registered to provide full chemical profiling (batch fingerprints and marker quantitation) as supplementary materials to enhance reproducibility.

### FDC operator training, credentialing, and fidelity control :

All Fire Dragon Cupping (FDC) sessions were delivered by licensed TCM nursing/therapy staff. Before independent practice, operators completed an institutional training program that included didactics (indications/contraindications, acupoint mapping, heat safety), hands-on practice, and supervised cases; competency was confirmed with a checklist and a minimum of ~50 supervised/simulated procedures. A written SOP harmonized acupoint mapping and sequence, session duration (back 15–20 minutes; abdomen ~20 min), and walking-cup repetitions, as well as heat control using dual criteria: patient-reported comfortable warmth without pain and infrared-thermometer targets of ~40–43 °C at the skin surface. The number of moxa pillars and replacement frequency followed the SOP to maintain stable thermal output. To ensure fidelity, operators documented each session with a one-page checklist (key steps completed, acupoints addressed, session duration, thermal readings, patient tolerance) and recorded any adverse events. A senior TCM practitioner audited randomly sampled sessions and convened monthly calibration meetings to review deviations and reinforce standards. In this study, “FDC” refers to Fire Dragon Cupping (not fixed-dose combination therapeutics).

### Outcome measurements:

The primary outcome was the change in TCM syndrome score after three months of treatment. The TCM syndrome scoring in this study followed the National Administration of TCM’s “Clinical Terminology of Traditional Chinese Medical Diagnosis and Treatment” (1997) and the “Guidelines for the Diagnosis and Treatment of Common Diseases in TCM Gynecology,” which specify item structure, anchor definitions, and clinical applicability for dysmenorrhea.^9^,^14^ These authoritative documents have been widely adopted in clinical and research settings and were used to ensure methodological consistency in our assessment of CCBS-related symptoms. According to the “Guidelines for the Diagnosis and Treatment of Common Diseases in TCM Gynecology”, 17 items were evaluated with the following scoring: five points for mild abdominal pain before and after menstruation (basic score), one point for unbearable abdominal pain, 0.5 points for obvious abdominal pain, one point for restless sitting, two points for shock, 0.5 points for pale complexion, one point for cold sweat, one point for cold limbs, one point for bed rest, one point for affecting work and study, one point for not relieving with general pain relief measures, 0.5 points for temporarily relieving pain with general pain relief measures, 0.5 points for lower back pain, 0.5 points for nausea and vomiting, 0.5 points for anal prolapse and 0.5 points for pain within one day (0.5 points added for each additional day). A higher total score indicated more severe menstrual pain.

The secondary outcomes included changes in pain levels before and after treatment, uterine artery hemodynamics and changes in endothelin-1 (ET-1) and nitric oxide (NO) levels. Pain was measured using the Visual Analog Scale (VAS) standard, where no pain is scored 0 points, mild pain is scored 1-4 points, moderate pain is scored 5-7 points and severe pain is scored 8-10 points.^15^ The VAS is a widely validated instrument with established reliability, validity, and responsiveness for clinical pain assessment, including gynecologic pain.^5^,^15^ Uterine artery hemodynamics were assessed using color Doppler ultrasound (Philips HDI-5000, Netherlands), including the resistance index (RI) and pulsatility index (PI).

ET-1 and NO levels were measured in the serum of 4-ml of peripheral venous blood by radioimmunoassay and nitrate reduction method, respectively. The reagent kits were purchased from Beyotime (Shanghai, China).

### Statistical analysis:

Data analysis were conducted using SPSS 22.0 (SPSS Inc., Chicago, Illinois, USA) and PRISM8.0 software (GraphPad, San Diego, USA). Classification data, such as education level and marital and reproductive history, were presented as frequency or percentage. Continuous data, such as age and disease duration, were reported as mean ± standard deviation (SD) or median and interquartile range (IQR) based on the distribution normality, as evaluated by the Shapiro-Wilk test. Normal distribution data were represented by mean ± standard deviation, an independent sample t-test was used for intergroup comparison and a paired t-test was used for intra-group comparison before and after the treatment. Non-normally distributed data were represented by median and interquartile range. The Mann-Whitney U test was used for intergroup comparisons and the Wilcoxon signed rank test was used for intragroup comparisons. Count data were analyzed using the chi-square test. A P-value of less than 0.05 (two-sided) indicated a statistically significant difference with a 95% confidence interval (CI).

## RESULTS

There was no statistically significant difference in basic clinical characteristics such as age, disease duration, age of menarche, body mass index (BMI), education level and marital and reproductive history between the two groups (all *P*>0.05) (Table-I).

**Table-I:** Comparison of Basic Clinical Characteristics between Two Groups.

Characteristics	FDC & SFZYD group (n=58)	SFZYD group (n=54)	t/Z/χ2	P
Age (years), mean±SD	25.07±3.76	25.63±4.54	-0.714	0.477
Disease duration (years), M(P25/P75)	2 (2-3)	2 (2-3)	-0.554	0.58
Age of menarche (years), M(P25/P75)	12 (12-14)	13.5 (12-15)	-1.537	0.124
BMI (kg/m^2^), mean±SD	22.15±2.41	21.32±2.17	1.914	0.058
***Educational level, n (%)***				
Junior high school and below	32 (55.17)	35 (64.81)	1.082	0.298
High school and above	26 (44.83)	19 (35.19)		
Marriage and childbearing history (yes), n (%)	21 (36.21)	23 (42.59)	0.478	0.489

***Note:*** FDC = Fire dragon cupping; SFZYD = Shaofu Zhuyu decoction; BMI = body mass index.

**Fig.1:**
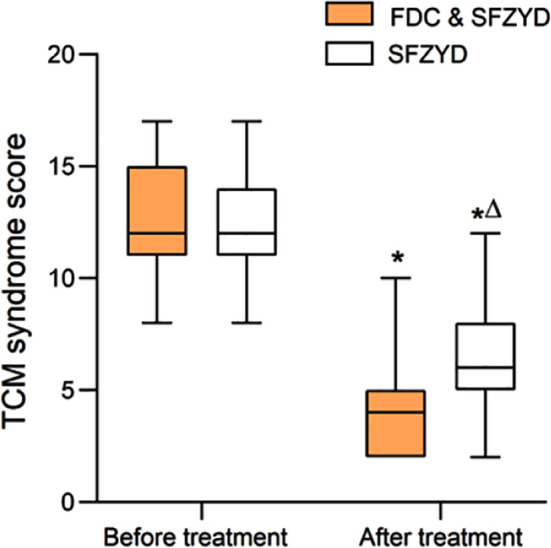
Comparison of TCM syndrome points between two groups; Compared with before treatment in the same group, *P<0.05; Compared with the FDC & SFZYD group, ^∆^*P*<0.05. FDC = Fire dragon cupping; SFZYD = Shaofu Zhuyu decoction; TCM = traditional Chinese medical.

Before treatment, there was no significant difference in the TCM syndrome scores of the two groups (*P*>0.05). After treatment, the TCM syndrome scores of both groups significantly decreased and were markedly lower in the FDC & SFZYD group than in the SFZYD group (*P*<0.05). Fig-1.

**Fig.2:**
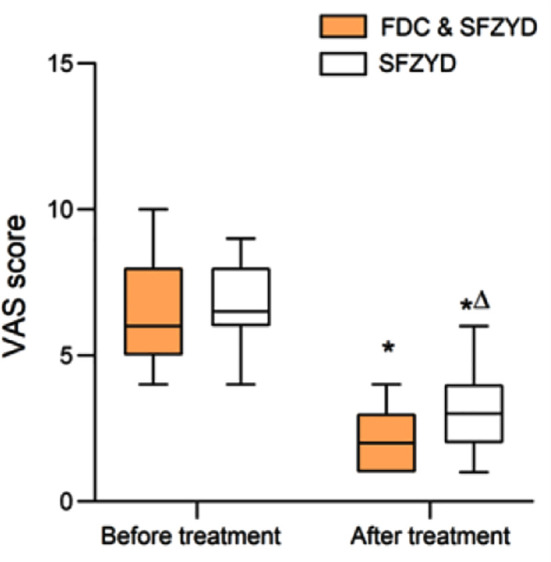
Comparison of VAS scores between two groups; Compared with before treatment in the same group, *P<0.05; Compared with the FDC & SFZYD group, ^∆^*P*<0.05. VAS = visual analogue scale; FDC = Fire dragon cupping; SFZYD = Shaofu Zhuyu decoction; TCM = traditional Chinese medical.

As shown in Fig.2, the pre-treatment VAS scores of the two groups were comparable (*P*>0.05). After treatment, the VAS scores of both groups decreased significantly compared to before treatment and were considerably lower in the FDC & SFZYD group (*P*<0.05).

**Fig.3:**
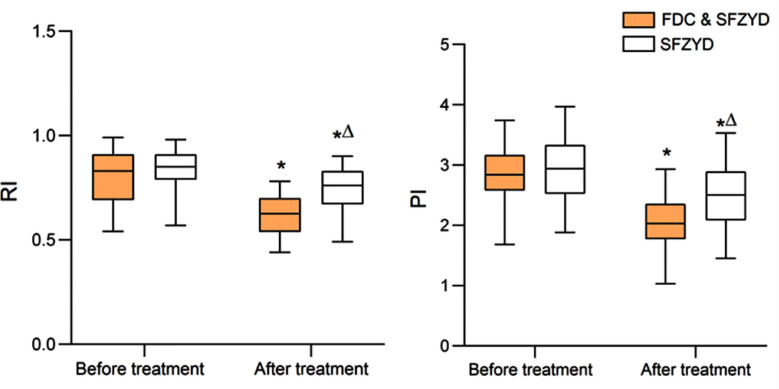
Comparison of hemodynamic status of uterine arteries between two groups; Compared with before treatment in the same group,*P<0.05; Compared with the FDC & SFZYD group, ^∆^*P*<0.05. FDC = Fire dragon cupping; SFZYD = Shaofu Zhuyu decoction; TCM = traditional Chinese medical. RI = resistance index; PI = pulsatility index; both are dimensionless indices.

As demonstrated in Fig.3, there was no significant intergroup difference in the pre-treatment RI and PI (both *P*>0.05). Post-treatment RI and PI of both groups decreased compared to before treatment and were significantly lower in the FDC & SFZYD group compared to the SFZYD group (both *P*<0.05).

**Fig.4:**
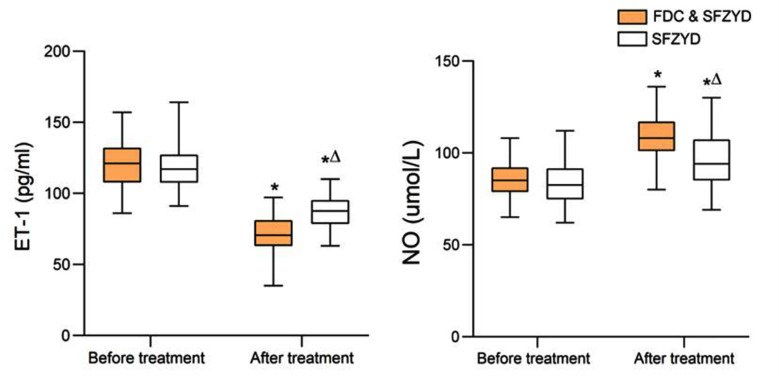
Comparison of ET-1 and NO levels between two groups; Compared with before treatment in the same group, *P<0.05; Compared with the FDC & SFZYD group, ^∆^*P*<0.05. FDC = Fire dragon cupping; SFZYD = Shaofu Zhuyu decoction; TCM = traditional Chinese medical; ET-1 = endothelin-1; NO = nitric oxide.

Serum ET-1 and NO were similar in the two groups before the intervention, as shown in Fig.4 (P>0.05). Both interventions led to a marked decrease in the ET-1 levels and an increase in the levels of NO (*P<0.05*). However, the combined FDC & SFZYD treatment was associated with significantly lower levels of ET-1 and higher levels of NO compared to the SFZYD treatment alone (both *P*<0.05).

## DISCUSSION

This study found that in PD patients with CCBS, the combined FDC and SFZYD treatment was more effective in lowering TCM syndrome scores, alleviating clinical symptoms, reducing pain and regulating hemodynamic status than SFZYD alone. According to the TCM theory, the treatment of dysmenorrhea caused by CCBS should be based on the principles of warming the meridians, dispelling cold and promoting blood circulation to remove blood stasis.^3^,^4^,^16^ The results of this study confirm previous observations that FDC and SFZYD are effective treatment options for PD. The studies of Zhong et al.^11^ and Wang et al.^17^ have shown the effectiveness of FDC in treating PD. A meta-analysis by Lee et al.^18^ also showed that SFZYD treatment is superior to conventional drug therapy for PD. FDC can achieve meridian dredging, meridian circulation and blood circulation through scraping, massage, etc. Moreover, its warming effect can also regulate the meridians, promote qi circulation and activate blood circulation.^10^,^11^,^19^

The components of the SFZYD preparation, such as Chuanxiong rhizoma (Chuanxiong), Corydalis rhizoma (Yanhusuo), Typhae pollen (Puhuang) and Trogopterori Faeces (Wulingzhi), which can promote blood circulation, regulate qi and relieve pain; Angelicae sinensis radix (Dangui) and Paeoniae radix rubra (Chishao) can promote blood circulation and remove blood stasis; Cinnamomi cortex (Rougui), Foeniculi fructus (Xiaohuixiang) and Zingiberis rhizoma (Ganjiang) are used to warm the blood vessels, promote blood circulation and regulate qi. The thermal-mechanical effects of FDC, together with the bioactive constituents of SFZYD, may synergistically promote blood flow, attenuate pain, and exert warming, cold-dispelling actions.^12^,^13^,^18^ The results of this study further emphasize that the synergistic action of FDC and SFZYD can achieve higher benefits in treating PD.

This study demonstrated that the RI and PI in patients who received FDC combined with SFZYD were significantly lower than in patients treated with SFZYD alone. This result indicates that the FDC and SFZYD regimen is more effective in improving the blood flow status of patients. Evidence suggests that SFZYD can effectively adjust the levels of RI and PI indicators in patients and alleviate clinical symptoms of PD.^20^ An animal study by Chen et al.^21^ showed that SFZYD may regulate the abnormal expression of pro-inflammatory cytokines and anti-inflammatory cytokines through mitogen- and stress-activated protein kinase 1/2(MSK1/2), improving the symptoms of dysmenorrhea in rats with CCBS syndrome. Zhang et al.^22^ also confirmed that SFZYD can promote blood circulation, remove blood stasis, warm and unblock the uterus, dispel cold and dampness, reduce swelling and nodules, regulate qi and relieve pain, effectively alleviating the clinical symptoms and pain level of patients with PD and improving the overall intervention effect. A study by Zhong et al.^11^ demonstrated that the combination of ibuprofen sustained-release capsules and FDC in the treatment of PD resulted in more significant improvements in RI and PI levels, which is consistent with the results of this study. From a physiological perspective, uterine artery RI and PI are Doppler-derived, dimensionless indices reflecting downstream vascular resistance; decreases in these indices indicate improved perfusion and reduced myometrial spasm, which are pathophysiological hallmarks of primary dysmenorrhea.^21^ Consistent with this interpretation, our post-treatment changes in vasoactive mediators (ET-1↓, NO↑) also support a shift toward lower vascular tone.^22^,^23^ In view of the retrospective design and the absence of synchronized repeated-measure acquisition of RI/PI and VAS across all cycles, as well as the limited sample size, we intentionally refrained from conducting post-hoc correlation analyses to avoid overinterpretation. Future prospective, multicenter studies will pre-register correlation and mediation analyses (e.g., testing whether changes in RI/PI and ET-1/NO mediate pain reduction) under standardized measurement protocols.

Previous studies showed that the symptoms of dysmenorrhea are closely related to the levels of serum NO and ET-1.^24^ As a vasodilator, NO can dilate blood vessels and regulate local vascular microcirculation.^23^ ET-1 is mainly expressed in uterine tissue and can exert significant vasoconstrictive and smooth muscle effects.^24^ In this study, the levels of ET-1 and NO in the FDC & SFZYD group were superior to those in the SFZYD group after the treatment. This result indicates that the combination of the FDC and SFZYD can more effectively regulate the levels of ET-1 and NO in PD patients with CCBS compared to SFZYD monotherapy. It is possible that the FDC therapy may improve local blood circulation, promote vascular dilation, increase blood flow, alleviate uterine ischemia and spasm and regulate the production of ET-1 and NO.^25^ At the same time, SFZYD can regulate the circulation of qi and blood in the body, improve the condition of uterine coldness, alleviate symptoms of blood stasis, regulate the function of vascular endothelial cells, reduce the secretion of ET-1 and increase the release of NO. To the best of our knowledge, this is the first study that provides evidence on the effect of FDC combined with SFZYD on the levels of ET-1 and NO in patients.

The study’s results demonstrated that the combination of FDC and SFZYD is safe, effective and feasible for treating PD patients with CCBS, providing a practical reference and guidance for the treatment of this patient group.

In this study, patients with primary dysmenorrhea of cold coagulation and blood stasis (CCBS) type were treated with Fire Dragon Cupping (FDC) combined with Shaofu Zhuyu Decoction (SFZYD), and outcomes were assessed across complementary domains, including patient-reported symptom scores, uterine artery hemodynamics, and serum vasoactive mediators. Compared with SFZYD alone, the combined regimen was associated with greater pain reduction accompanied by measurable improvements in blood-flow indices and biochemical markers, suggesting a consistent trend between clinical benefit and objective physiological changes. The integration of subjective, imaging-based, and laboratory outcomes, together with standardized intervention procedures and a relatively homogeneous syndrome population defined by explicit diagnostic criteria, enhances the internal consistency and interpretability of the findings. Nevertheless, as a retrospective single-center analysis, the results should be interpreted cautiously, and further validation is required. Prospective, multi-center randomized controlled trials with longer follow-up would help clarify the durability of benefit and recurrence risk, while embedding systematic adherence and fidelity monitoring to better verify treatment exposure. Additional work could include stratified analyses by CCBS subtype and pre-specified correlation or mediation models to test whether changes in hemodynamic and biochemical parameters mediate symptom improvement. Incorporating comprehensive chemical profiling of SFZYD batches and structured safety surveillance, such as CTCAE-based grading, would further strengthen reproducibility and facilitate generalizability to broader clinical settings.

### Limitations:

This study has several limitations. It was a single-center retrospective analysis with a relatively small sample size, which may limit generalizability. The proper administration of FDC requires specialized training, and objective fidelity metrics (e.g., temperature curves, pressure logs, inter-operator agreement) were not systematically recorded. Future prospective trials should incorporate sensor-based monitoring, pre-specified fidelity thresholds, and standardized training to ensure operator consistency. The follow-up period covered only three menstrual cycles, preventing assessment of long-term efficacy, recurrence, and safety; prospective multicenter randomized trials with at least 6–12 months of follow-up are warranted. Only patients with CCBS syndrome were included, and no standardized tool was available to classify subtypes (predominantly cold vs. blood stasis), precluding subgroup analyses; future studies will incorporate standardized subtype assessment and adequate sample sizes for powered comparisons. Because grouping was based on actual treatment received, selection bias cannot be excluded, and although baseline characteristics were similar, more severe cases may have been more likely to choose combination therapy. Detailed adherence data and standardized attendance logs were unavailable, hindering calculation of completion rates; future studies should use predefined adherence thresholds and electronic attendance monitoring. Safety data were limited, as minor adverse events may have been underreported; standardized adverse-event surveillance and grading (e.g., CTCAE or TCM-adapted scales) should be implemented in future studies. Additionally, while pharmacopoeial QC and HPLC fingerprinting are routinely performed, complete batch-level chromatograms were not archived; future trials should pre-register full chemical profiling and provide supplementary batch data to enhance reproducibility. Finally, correlation analyses between RI/PI and pain scores were not performed due to the lack of synchronized repeated-measure data; future trials should pre-register adequately powered correlation and mediation analyses under standardized protocols.

## CONCLUSION

Compared with the SFZYD treatment alone, the combination of FDC therapy and SFZYD can more effectively reduce the pain level of PD patients with CCBS, alleviate clinical symptoms, regulate the hemodynamic status of uterine arteries and the levels of ET-1 and NO.

### Authors’ contributions:

**ZY:** Study design, literature search and manuscript writing.

**SZ, XL, ZQ, WB and YZ:** Data collection, data analysis and interpretation. Critical Review.

**ZY:** Manuscript revision and validation and is responsible for the integrity of the study.

All authors have read and approved the final manuscript.
